# How to prepare nursing students for mental health clinical engagement: a qualitative study

**DOI:** 10.1186/s12909-023-04657-8

**Published:** 2023-09-18

**Authors:** Maryam Shaygan, Azita Jaberi, Fahimeh Alsadat Hosseini, Malek Fereidooni Moghadam

**Affiliations:** 1 Community Based Psychiatric Care Research Center, Shiraz University of Medical Sciences, Shiraz, Iran; 2 Nursing Department, Shiraz University of Medical Sciences, Shiraz, Iran

**Keywords:** Mentally ill patient, Nursing students, Clinical placement, Qualitative study

## Abstract

**Background:**

Since clinical experience is challenging, identifying the factors influencing the learning process and acquiring clinical competence in mental departments is essential. Limited studies have investigated students’ concerns regarding attending this clinical setting and how they are mentally and academically prepared.

**Aim/question:**

Explaining the various aspects of nursing students' preparation to attend the mental clinical environment.

**Methods:**

This qualitative study was conducted on bachelorette nursing students and college professors using in-depth, semi-structured interviews. Inductive content analysis was used for data analysis, and Lincoln and Guba’s criteria were used for the rigor of the data.

**Results:**

The participants’ viewpoints regarding how to prepare nursing students to enter mental clinical settings can be summarized in 4 categories: “understanding the students’ concerns” “understanding the students’ expectations” “the necessity of the students’ mental preparation” and “preparing the scientific materials needed to attend in a mental ward”.

**Conclusion:**

Nursing students have fears and worries about entering mental departments and have expectations of themselves and their instructors. To help students deal with these concerns, psychological and educational preparations should be provided, among which the role of new educational technologies can be mentioned.

## Accessible summary

- What is known about aboutthe subject?
  Clinical training is the main and most important part of nursing education programs, so students may experience tension, fear, or high levels of stress and anxiety, as well as feelings of helplessness and the fear of making mistakes before clinical training in mental wards.
- What the paper adds to existing knowledge.
  The results showed that the students have fears and worries about entering these departments and have expectations from themselves and their instructors. Also, the findings indicated that to help students to deal with these concerns, psychological and educational preparations should be provided.
- What are the implications for practice?
  In addition to the scientific and skill preparation of nursing students for mental health care engagement, urgent measures are necessary to change their negative attitude regarding these settings.

## Introduction

Clinical training is the main and most important part of nursing education programs; that is, more than half of nursing students’ training course is clinical training [1]. Therefore, providing rich and favorable clinical experiences is very important in nursing education [2]. These clinical experiences lead to the formation of students’ attitudes towards the nursing profession and, as a result, have a direct impact on their career choice and even their responsibility in the clinical environment [3]. Nevertheless, clinical education in nursing has always been associated with many challenges [4, 5]. The most important challenge for educators is to improve the ability of undergraduate students to identify and respond appropriately to issues in clinical settings [6].

Among all clinical training environments in nursing, training in mental wards may be one of the most difficult experiences [7]. Undergraduate students often enter the mental health course with a preconceived image of patients that is influenced by the media and a lack of proper knowledge about the patients’ conditions, which is exacerbated by the attitudes of their families. Therefore, students may experience tension, fear, or high levels of stress and anxiety [8] and feelings of helplessness and fear of making mistakes [9, 10], before clinical training in mental wards. This issue can cause students’ stress during clinical practice and lead to negative experiences and attitudes toward mental health nursing, consequently preventing them from having a comfortable and informative experience [11] and even leading them to avoid choosing psychiatric nursing as a career [12].

On the contrary, if clinical education is perceived positively, nursing students develop and acquire new knowledge and skills about how to deal with people suffering from mental illness. This, in turn, leads to an increase in self-confidence and a decrease in their worries and fears, and as a result, they become more willing to work as a mental health nurse [13]. Therefore, to effectively improve the negative attitude of nursing students towards these patients and clinical settings, appropriate training in this field is of vital importance [11, 14]. Since clinical experience is inherently challenging, identifying the factors influencing the learning process and acquiring clinical competence in mental departments, and specifically, identifying the previous backgrounds of students in these settings and creating scientific and psychological preparation in them is an educational prerequisite.

Up to now, various studies have investigated the attitudes and viewpoints of nursing students towards mentally ill patients and their care, and the results of these studies range from positive to mostly negative attitudes amongamong students [3, 6, 7]. However, limited studies have investigated students’ concerns regarding attending this clinical setting and how they are psychologically and academically prepared. Therefore, this study was conducted with the aim of “explaining the various aspects of nursing students’ preparation to attend the mental clinical environment”.

## Methods

### Study design

We used a qualitative research method with a conventional content analysis approach according to the Granheim & Laundman method [15]. The conventional content analysis method is usually appropriate when the existing theories or research literature on a phenomenon is limited and the researchers immerse themselves in data to uncover new insights [16]. This study followed the Consolidated Criteria for Reporting Qualitative Research (COREQ) reporting guideline for qualitative studies.

### Participants and setting

The present study was conducted from December 2021 to October 2022 at Shiraz University of Medical Sciences (Grant code: 24,921). In Iran, based on nurse education curriculum, Bachelorate degree in nursing is a 4-year course, in which, nursing students have been provided 34 h theoretical preparations regarding mental health in their 6th semester (3rd year) and then they enter to the clinical setting with the presence of a well-trained college teacher. During this eight-week clinical practice, the students learn about various mental health conditions and how to communicate and manage them under the supervision of the teacher.

Participants of the present study were 3rd year bachelorette nursing students and college professors, purposefully recruited. The inclusion criteria for students included: a desire to participate in the study, studying at the undergraduate level of nursing, students who were about to attend in the mental ward or had recently completed their attendance, having no previous experience working in this field, not suffering from chronic mental illnesses (according to the student’s own report), the ability to share rich information about the subject of study, and the ability to speak Persian. The inclusion criteria for instructors were: willingness to participate in the study, a history of studying in the field of mental and working in the mental clinical setting as a trainer and lecturer for at least 1 year. The selection of participants and conducting of interviews continued until data saturation was reached, that is, the data had become repetitive and no new data were obtained. By conducting 14 interviews, data saturation occurred, no participant left the study, and there was no need to re-interview any of the participants.

### Data collection

To collect data, individual, in-person,semi-structured interviews were used.The interviewers (AJ and MSh) were both female and experienced with qualitative methods and had PhD in nursing education, piloted the interview guide. One of the interviewers (MSh) was professionally involved in this course with students as a mental health college professor, which her background could help with the interpretation of the data. The second interviewer (AJ) was a college professor experienced in qualitative research for dissertion projects. She also had previous experience of publishing qualitative articles in peer review journals.

The interview sessions started with an explanation of the study’s purpose and obtaining written consent by the first and corresponding authors. The interview questions were focused on the main purpose of the research (explaining the various aspects of the preparation of nursing students to attend the mental wards). Each interview began with an interview guide, followed by probing questions to clarify and elicit further information, such as “What do you mean by that”“?”“Can you give an example”“?”“Can you explain more?” The interview guide questions are provided in Table 1.

**Table 1:** The interview guide questions

1- In your opinion, what do the students who are going to attend the mental health setting have in mind and how do they think about this department?
2- How did you feel the first day you entered the ward and what did you think about the patients of these departments?
3- What idea do you have in your mind about this hospital and hospitalized patients?
4- In your opinion, what preparations or requirements should be provided to you in advance to attend these clinical settings?
5- In your opinion, what training or skills should be taught to you before entering these wards?
6- In your opinion, what training methods or arrays are more suitable for better preparation to attend these departments and how should they be used?

The time and place of the interview were determined with the coordination of the participants, and the interview was conducted whenever they were comfortable, without presence of anyone else. At the end of each session, the interview was summarized by the researcher, who made an appointment for future sessions if needed. The participants were also informed that the research team might contact them for a “member check” after verbatim transcription. The duration of the interviews was between 45 and 60 min. Each interview session was audio recorded and then transcribed verbatim into Farsi immediately.

### Data analysis

To analyze the data in this study, inductive content analysis was used, that is, the data derived from the codes and interviews. In this method, first, the text of the interviews was written down word for word, which formed the analysis unit. After reading the interviews several times to get a general impression, the meaning units were determined in the form of sentences or paragraphs by first and corresponding authors (MSh & AJ), and after they were condensed, primary or open codes were extracted. Afterward, the codes were categorized into subcategories and categories based on similarities and differences [17]. Data management was performed by Microsoft OneNote software for Windows XP.

To obtain the trustworthiness of the data in the present study, the criteria of Lincoln and Guba [18] were used as follows: 1- Credibility was ensured via prolonged engagement with the study, immersion in the data, peer checking by experts in qualitative studies and mental health education, and member checking with participants. 2- Transferability was ensured through providing detailed information about the study and its findings so that other researchers in the area of mental health clinical setting engagement can use the findings of the study as a source of information. 3- Dependability was ensured via an audit trail with detailed descriptions about data collection, interview guide, data analysis, and participants’ quotes.

### Ethical considerations

The Ethics Committee of Shiraz University of Medical Sciences, Shiraz, approved this study (code IR.SUMS.NUMIMG.REC.1400.079). Permission for data collection was obtained from the participants and the authorities of the study setting. Moreover, informed consent was obtained from participants. Participation in the study was voluntary, and participants were able to withdraw from it at will and assured of confidentiality.

## Results

Semi-structured in-depth interviews were conducted with 14 participants (12 students and 2 professors) in this study. The mean age of the participants was 26.23 ± 8.14 years, and they were in the age range of 21 to 45 years. The demographic characteristics of the participants are presented in Table 2. The analysis of the data showed that the participants’ viewpoints regarding how to prepare nursing students to enter mental clinical settings can be summarized in 4 categories: “understanding students’ concerns” “understanding students’ expectations” “necessity of mental preparation of the student” and “preparing the scientific materials needed to attend in a mental ward” (Table 3).

**Table 2:** The participants’ demographic characteristics

Participant no.	Age (years)	Gender	Academic year	Attended in mental ward
Nursing students				
P1	24	Male	4	Yes
P2	22	Female	4	Yes
P3	23	Female	4	Yes
P4	22	Male	4	Yes
P5	22	Male	4	Yes
P6	24	Female	3	No
P7	25	Male	4	Yes
P8	23	Male	3	No
P9	21	Male	3	No
P10	25	Female	3	No
P11	24	Male	4	Yes
P12	30	Female	4	Yes
College professors	Age	Gender	Work experience	
P13	42	Female	10	
P14	45	Male	14	

**Table 3:** Codes, subcategories and categories emerged from data analysis

Codes	Subcategories	Categories
Fears of patients becoming uncontrollable Fears of unknown environment Fears of being attacked by patients	Recognition of fears and worries	Understanding students’ concerns
Feeling despair and hopelessness Feeling of being in a prison Feeling sad about patients’ problems	Recognizing sadness	
Expecting the instructor to be the supporter Expecting the instructor to be an educator	Students’ expectations from the instructor	Understanding students’ expectations
Desire to know themselves Capability to communicate with patients Having empathic relationship instead of sympathy Ability to establish trust	Students’ expectations from themselves	
Encouraging students for scientific preparation to attend in mental ward Encouraging students to obtain practical skills Encouraging students to express their fears and concerns	Overcoming fears and worries	Necessity of mental preparation of the student
Providing evidence-based information about the mentally ill patients Assessment and correction of the misconcepts regarding these patients Expressing the positive experiences and memories	Establishing the right attitude towards the patients of mental departments	
Necessity of providing educational materials regarding effective communication skills Necessity of providing educational materials regarding patients’ assessment Necessity of providing educational materials regarding common psychiatric diseases, their signs and symptoms, and their management Necessity of providing educational materials regarding the hospital, equipment and setting	Necessity of remembering the basic concepts	Preparation of scientific materials needed to attend in mental ward
Necessity of providing educational movies regarding communication with patients Necessity of providing educational movies regarding patients’ assessment Necessity of providing educational movies regarding the clinical environment	Teaching practical skills	
Necessity of using SP to teach about the patient assessment and interview Necessity of using role playing to teach how to interview Necessity of using new educational technologies to achieve better learning	How to prepare the required scientific materials	

### Understanding students’ concerns

One of the main points that should be taken into consideration by the trainers when preparing students to attend the mental department is to know the fears, worries, and sadness the students face in these departments.

#### Recognition of fears and worries

Facing the clinical environment as aa nursing student is always accompanied by fears and worries due to its unknown nature. This issue is more challenging in mental departments because students are so worried about entering these departments based on their assumptions about these patients. They consider this ward a terrifying environment because they think that patients may have uncontrollable behaviors, so they may even attack students. In this regard, some students mentioned:

“*I thought that the patients in these departments were very aggressive and nervous and would attack us.” P3, nursing student*.

*“In addition, the unpredictability of the patients’ condition causes stress.” P1, nursing student*.

*“Those of us who have completed this internship feel that the environment was like a prison so that even a healthy person may go crazy there*.”*P2, nursing student*.

Of course, some students are also worried about not being able to establish a proper relationship with the patients and, for this reason, creating problems for themselves, the patients, and other members of the treatment team. One of the students stated:

*“I don’t know anything;; what should I answer to this patient?? how should I start the interview??and what should I say?” P2, nursing student*.

#### Recognizing sadness

In addition to the fears related to facing the mental health department, the students who recently attended the mental hospital experienced other concerns. Some of these students were struggling with feelings of hopelessness. They were saddened by observing the condition and problems of these patients, and sometimes they transferred this sadness caused by the patients’ problems to the family environment in such a way that it overshadowed their personal lives and they suffered disorders such as sleep disorders. In this regard, one of the participants stated:

“*When I enter this ward, I think about what problems brought them here.That’s why I am impressed, and I want to help them. Moreover, I feel sad and upset, and sometimes I feel distressed*.”*P13, college professor*.

### Understanding students’ expectations

For students to be placed in the right place in the mental ward, they also need to know what is expected of them in this field. This issue, raised in the statements of the participants, indicated that the students had expectations from the instructors and themselves and that by fulfilling them, the encounter with the patients and mental departments would be facilitated.

#### Students’ expectations from the instructor

One of the most important facilitators of clinical education is clinical trainers and educators. Students expect the instructors to play a strong role as their supporter in various situations in addition to the task of teaching, which was seen in the statements of the students as follows:

“*Because these patients are different from patients in other departments, I feel afraid. That’s why the teacher must pay attention to us at every moment. Especially during the first days. At the same time, to prepare us, both at the beginning and in the new meetings, they should orient us with the things that we are going to face.”P5, nursing student*.

*“I try to teach my students information about how to deal with these patients so that they don’t have any problems communicating, and I also try to be present by their side so that they feel that if something happens, I will support them. In addition, giving them enough information can reduce their anxiety.”P13, college professor*.

#### Students’ expectations of themselves

One of the interesting findings in the present study was the students’ expectations of themselves. The students who were previously present in this clinical environment said that in this department, people can get to know themselves better, and in fact, knowing more about themselves is one of the expectations that can be reached at the end of attending this department.

“*Self-knowing is one of the things that might happen for you in these wards and may change your life, and even motivate you.” P6, nursing student*.

*“Attending this clinical setting teaches a series of things about life … you learn how to build yourself and what skills to cultivate in yourself to stay safe from these diseases.” P13, college professor*.

The students also said that by taking this clinical course, they learned not to cry with the patients but to understand what they were going through. In this way, for the students who are going to work in these departments, one of the expectations is to learn empathy for patients.This issue is stated as follows:

*“I had a patient that I got very close to. I mean, I used to talk to her during my clinical course, and I even told her about my private life issues, but later I realized that this was a mistake and I sympathized with him instead of empathizing, and it even affected my own life.” P7, nursing student*.

In addition, the students stated that one of their expectations from themselves at the end of this training course is to be able to establish effective communication with patients, gain their trust, and then be able to provide them with the correct training.

*“Interviewing a mentally disturbed person is one of the things I believe we should learn … For example, how to ask questions or give them medicine.” P5, nursing student*.

### The necessity of mental preparation for the student

Based on what was mentioned in the previous categories, it is necessary to set up preparations to overcome fears and worries and to meet expectations, one of which is mental preparation. This includes taking measures to overcome fears and worries and creating creating a positive attitude towards towards the patients of these departments.

#### Overcoming fears and worries

To help students overcome their fears and worries, it is necessary to encourage students to express selvesthemselvesto determine what aspects of being in these departments caused fear and worry. One of the participants stated in this regard:

*“One of the things we ask our students to do during and after their clinical course is to talk about each of their patients. How they started, what feelings they had, what problems they had in the interview and communication. Overall, we try to have a reflection on this matter so that both their worries decrease and they learn more.” P14, college professor*.

In the next step, students can be encouraged to overcome these concerns by increasing their scientific knowledge as well as improving practical skills such as effective communication. This topic was mentioned in the interviews:

*“In terms of mental preparation, they should have a mental background about these patients so that they don’t have any fear of entering these departments. We (instructors) have to warn them about the precautions so that they feel reassured. When the students are anxious, their learning will reduce, and they cannot enter the environment and communicate with the patient.” P13, college professor*.

Other techniques that can help students reduce the fear of facing these patients are muscle relaxation techniques or stress management skills, anger management, assertiveness, and positive thinking, which was more evident in the statements of nursing instructors:

*“I give a series of workshops for my students, such as stress management, anger management, positive thinking, assertiveness, and communication skills, and when we want to enter the mental department, we review it again together. In my opinion, these are useful both for dealing with these patients and for the students’ personal lives.” P13, college professor*.

#### Establishing the right attitude toward the patients of mental departments

Considering that the negative attitude towards the mental departments is an important factor in causing panic, creating a positive attitude in students is one of the most important ways to reduce and overcome fear and worry. To achieve this goal, the participants believed that providing evidence-based scientific information about these patients could change and improve students’ attitudes.

*“All students have a negative image of mental patients, which makes them refuse to go alone to the patient’s room. It is possible to provide explanations about the patients and the department so that the student can prepare for what he is dealing with and behave accordingly.” P10, nursing student*.

Some participants also said that it is important to find out what people think and believe about these patients and then correct them by giving them the right information. The unknown nature of the mental wards and society’s view of these patients and departments are the contributing factors causing fear.

*“Everyone has a negative opinion about these wards.” P4, nursing student*.

*“We must tell the students that it is true that they they will be affected by these patients, but they must learn what they can do to help them and make their lives better. If the students know the nature of the disease, they will probably be less emotionally affected, and this creates an emotional awareness for them.” P14, college professor*.

One of the solutions for correcting misconceptions and creating a positive attitude, from the point of view of some participants, was to express the personal experiences of instructors or other students from mental departments and patients. They believed that by mentioning positive experiences, tensions, and perceptions and preconceptions, those negative emotions would be reduced, and students could attend the ward with more peace of mind.

“*If I wanted to say something to the students, I would say that it was very different from what I thought .The patients were much calmer and liked to talk.” P11, nursing student*.

*“I present this experience to the students (so that they both learn and their anxiety is somehow reduced). My first experience when I was a student was with a girl suffering from psychosis, and because the professor was not with me and I did not know the basic principles of communication, the patient became very dependent on me.” P13, college professor*.

### Preparation of scientific materials needed to attend in the mental ward

In addition to mental preparation, providing students with scientific materials, including theoretical knowledge, recalling basic concepts, and practical skills, is one of the most important strategies in alleviating students’ concerns and preparing them to face these patients.

#### The necessity of remembering the basic concepts

A large number of participants pointed out that before entering the clinical setting, important and basic content should be recalled and repeatedly taught to students in theoretical classes. The contents that the training participants prioritized were related to common psychiatric diseases, signs and symptoms of diseases, how to evaluate patients, effective communication techniques with patients, and how to care for patients with various disorders.

*“In my opinion, it is possible to use a combination of several methods for training, for example, text and voice are better for training nurses to care for these patients.”P9, nursing student*.

*“Regarding the scientific prerequisites, well, students should know common mental diseases, their symptoms, diagnostic criteria, and things like that.”P12, nursing student*.

Some participants also thought that, since students had never been in a clinical setting before, the training should also cover things like the physical environment of the hospital, the equipment and facilities of each department, comfort and recreation facilities, and what equipment patients are allowed to use.

*“It is necessary that they (teachers) provide us with a series of information about the atmosphere of the mental department in the form of images.”P2, nursing student*.

*“The student must have some information about the environment. For example, the rooms do not have doors;; there are isolated rooms in these wards, and there are no switches and sockets. How the windows, floor, and walls are. What treatment facilities exist or should exist, such as occupational therapy, group therapy, and a suitable environment for interviews.”P13, college professor*.

*“For example, we should tell them the definition of the department, the nature of the department and the patients and the difference between this department and other departments,and the process of hospitalization in the emergency, acute and special departments.”P14, college professor*.

#### Teaching practical skills

One of the important skills for nursing care, in addition to theoretical knowledge, are practical skills, including patient assessment and effective communication with patients in mental wards.

*“Communication skills are very important. Because for mental patients, the most important thing is talking and conversation.”P8, nursing student*.

*“(In my opinion) they (the students) should know the principles of interviewing patients in mental departments because it is completely different from other departments, and they should be familiar with communication techniques before entering the department.”P13, college professor*.

The participants believed that these skills are better learned using educational videos than textual content.

*“One of the problems of our education is that it is one-dimensional. It is better to use a combination of pamphlets, videos, applications, etc. so that the learning becomes less boring and the outcome increases.”P12, nursing student*.

*“Well, training with movies remains in the mind and is more effective than text materials. Especially, holding a meeting before the start of the clinical course could help us have a better perspective about this ward, the patients, and their symptoms.”P10, nursing student*.

*“Of course, if the educational contents have 3D images and animation in addition to the text content, it will become more attractive, or if we add sound and music to the 3D images.”P14, college professor*.

#### How to prepare the required scientific materials

One of the methods used by trainers to teach practical skills in mental departments is standardized patient (SP). For this purpose, one person trained for this subject, with a pre-determined scenario exercise, will perform one of the mental disorders or effective communication techniques in front of the students.

*“In my opinion, first of all, a workshop should be set up by the professor, and the professor should play the roles of patient and therapist with a standardized patient, and the students should observe and learn how to treat the relevant patient in this situation.”P9, nursing student*.

Another common method for training how to interview a patient is role-playing, which was also mentioned by the instructors and students in the present study. In this method, one of the students takes the role of the patient, and the instructor plays the role of the interviewer. In this way, students will practically observe the interview process and ask their questions in this field.

*“Because most of the work in these departments is communication and interaction, role-playing is the best way to teach it. For this purpose, scenarios must be written, some of which include communication mistakes, so that the student learns what not to do.”P2, nursing student*.

One of the key suggestions of the participants of the present study was to use new technologies to teach learning objectives. They believed that the design of web-based applications and software using scientific and approved scenarios regarding the evaluation and communication with these patients can prepare the students well to face these patients. However, students proposed conditions for these new technologies to increase their usability.

*“In my opinion, a set of methods should be used to teach skills for this ward. Videos, the experiences of trainers, simulations, and practical applications are some of them.”P8, nursing student*.

*“It is very good to provide the students with a series of training through mobile phones that they can use at all times.”P13, college professor*.

## Discussion

The current study, which was done to find out what nursing students and instructors thought about how to be scientifically and psychologically ready to go to the mental department, showed that the students have fears and worries about going there and have expectations for themselves and their instructors. Also, the results showed that students should be given both psychological and educational preparations, such as the use of new educational technologies, to help them deal with these worries. There are not many studies that qualitatively examine the scientific and psychological preparation of nursing students to attend the mental department. Most of these studies have dealt with the students’ experience of attending these departments [19], and the existence of fears and concerns in students is consistent with the findings of the present study. In one of these studies, the students explained the stress in the mental health department, both regardingcaring for patients and the way nurses behave [8].

Despite those fears, students were able to grow and develop their competencies in four stages: (1) breaking the stigma of mental illness, (2) establishing trusting communication with patients, (3) acquiring professional skills and knowledge, and (4) students acknowledging the importance of personal development [8]. As the results of one study showed, one of the basic problems in facing these patients from the students’ viewpoint was the stigma of these patients [20], which was shown in the form of fear and concern in the present study. The concern about not being able to communicate and not having enough knowledge and skills in this field is among the challenges faced by students in the study of Al-Showkan et al., as well [21]. In another study, anxiety, fear, and apprehension at the beginning of entering this department were also mentioned by the nursing students [22] and resolved eventually in the process of self-reflection [23]. Comparing the findings of the previous studies with the present study shows that nursing students struggle with stress and fear while attending these departments, partly due to aa lack of knowledge about these departments and patients and a lack of abilities and skills such as communication skills.

Another concern of the students regarding being in the mental department was the sadness caused by seeing the patients’ problems and not being able to resolve them. This experience and the stress of work in these departments may be such that it leads to depression in nursing students because they feel that these patients experience a lot of suffering and the students feel that they do not have enough time, knowledge, competence, and skills to communicate with them [24]. In addition, in some cases, instructors and supervisors limited students’ exposure to these patients, which led to the students’ dissatisfaction since they did not have much contact with patients [25]. Considering the findings of the aforementioned studies and the present study, it seems that by identifying interested students and using appropriate methods, instructors can increase the skills and competence necessary to care for these patients.

On the other hand, one of the advantages that the students gained from attending these departments was a better understanding of themselves, which is mentioned in other studies:“I learn a lot about the human being” [24]. Nursing students mentioned that being in this department has changed them so that they are no longer afraid of these patients and that it has created self-awareness in them. It means that it has made them more sensitive to other people’s feelings and communication skills [26]. In another study, the students also believed that the presence and care of mentally ill patients led to personal development [21]. The results imply that despite the many problems that students pointed to due to attending these departments, positive benefits such as self-knowledge are also expected.

One of the expectations that nursing students and nurses are expected to have in caring for patients is to establish proper communication, including empathic communication. Meanwhile, in existing studies, it has been seen that the level of empathy in nurses and other healthcare workers has decreased [26]. The use of communication skills, the presence of a safe emotional and physical environment, and continuous feedback from instructors to students can increase the ability to empathic communication [25], as can placement in different departments of the hospital and a supportive learning environment [27, 28], in which numerous and diverse clinical learning opportunities are available. Additionally, as the results of a systematic review showed, mental health nurses and educators are key people who can play an important role in creating a positive attitude in nursing students [29]. All of the above-mentioned cases include creating a safe learning environment, providing feedback from instructors, establishing a supportive learning environment, and the presence of instructors who create a positive attitude in students. Considering that the negative attitude towards mental departments is one of the reasons for the staff shortage in these departments [30–32], knowing these concerns and attitudes and using strategies to create a positive attitude in students can be a great help in solving the staff shortage in these departments. This strategy has also been mentioned in another study [29].

In addition to creating a positive attitude and mental preparation in students, scientific preparation was also another important issue that students and professors in the present study emphasized. Teaching and reviewing basic concepts and the use of diverse and practical educational methods, such as educational videos, role-playing, SP [33], and modern educational methods, i.e., new technologies, applications, or websites, were some strategies for student preparation and education. Advanced methods such as simulators were also one of the training methods; however, studies in this regard in mental health nursing education are scarce [34, 35]. In one of the studies where a low fidelity simulator was designed to communicate with patients suffering from mental disorders, it was observed that in addition to communication skills, students were able to learn decision-making skills and therapeutic interventions, and their reflection and critical thinking also improved [36]. In another qualitative study, it was also shown that mental health simulation has improved the experience of a placement in the clinical environment [37]. Since the use of new technologies is favored by young people, it seems that educational instructors should use new educational techniques in teaching and preparing students to attend the mental clinical environment.

Despite the difference between the studies conducted in this field in terms of methodology, the findings show that providing more training on mental health nursing and longer placement in these departments can help improve students’ attitudes. Creating the belief that nursing in these departments can have valuable effects on society and that the education of nursing students should be considered in the training sessions.

One of the most important strengths of the present study is that the findings provide baseline information regarding the necessary preparations to attend mental health departments that the instructors, educational authorities, and nurses of these departments need to know so that, by using it, they can improve the students’ attitudes by implementing appropriate measures. Since nursing students have not yet acquired the necessary competence and knowledge after graduation, they need guidance and support when they enter clinical departments.

### Implications for practice

In addition to the scientific and skill preparation of nursing students for mental health care engagement, urgent measures are necessary to change their negative attitude regarding these settings. The findings of the present study, especially in the field of educational methods such as movies, role-playing, and the use of mobile and web-based applications, can open the way for trainers to reduce students’ worries and concerns and subsequently create a positive attitude in them.

## Conclusion

Being in the mental department is associated with anxiety and worries that may disrupt the care of hospitalized patients. Therefore, these departments need interested nurses who have acquired the necessary knowledge, skills, and competency in this field. Providing care to these patients should be done by experienced and trained people with a positive attitude. Unfortunately, in recent years, the negative attitude toward mental health departments has increased in the nursing community. Therefore, in addition to scientific and skill preparation, urgent measures are necessary to change this attitude. The findings of the present study, especially in the field of educational methods such as movies, role-playing, and the use of mobile and web-based applications, can open the way for trainers to reduce students’ worries and concerns with their help and subsequently create a positive attitude in them.

## Data Availability

The datasets used and/or analyzed during the current study are available from the corresponding author on reasonable request.
